# Buccal and Sublingual Vaccines: A Review on Oral Mucosal Immunization and Delivery Systems

**DOI:** 10.3390/vaccines9101177

**Published:** 2021-10-14

**Authors:** Valeria Trincado, Rikhav P. Gala, Javier O. Morales

**Affiliations:** 1 Drug Delivery Laboratory, Departamento de Ciencias y Tecnología Farmacéuticas, Universidad de Chile, Santiago 8380494, Chile; valeria.trincado@ug.uchile.cl; 2 Advanced Center for Chronic Diseases (ACCDiS), Santiago 8380494, Chile; 3 Center of New Drugs for Hypertension (CENDHY), Santiago 8380494, Chile; 4 Biotechnology Division, Center Mid-Atlantic, Fraunhofer USA, Newark, DE 19702, USA; Rikhav.Gala@fhcmb.org

**Keywords:** mucosal immunity, buccal vaccines, sublingual vaccines, adjuvants, antigen delivery systems

## Abstract

Currently, most vaccines available on the market are for parental use; however, this may not be the best option on several occasions. Mucosal routes of administration such as intranasal, sublingual, and buccal generate great interest due to the benefits they offer. These range from increasing patient compliance to inducing a more effective immune response than that achieved through conventional routes. Due to the activation of the common mucosal immune system, it is possible to generate an effective systemic and local immune response, which is not achieved through parenteral administration. Protection against pathogens that use mucosal entry routes is provided by an effective induction of mucosal immunity. Mucosal delivery systems are being developed, such as films and microneedles, which have proven to be effective, safe, and easy to administer. These systems have multiple advantages over commonly used injections, which are simple to manufacture, stable at room temperature, painless for the patient since they do not require puncture. Therefore, these delivery systems do not require to be administered by medical personnel; in fact, they could be self-administered.

## 1. Introduction

**Figure 1:**
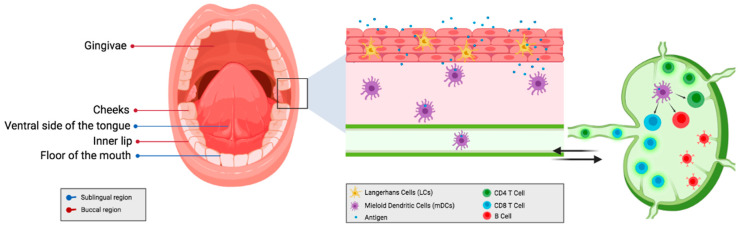
Antigen uptake and presentation by dendritic cells through the oral mucosal route for immunization.

The oral cavity is one of the most widely accepted and patient-friendly routes to administer a variety of drugs. The oral cavity includes the gingival areas, the back and ventral areas of the tongue, the cheeks, the inner lips, and the floor of the mouth (Figure 1). The oral cavity allows access to several routes of administration of medicines, the most used is the route that includes the gastrointestinal tract, where the drug passes through the esophagus into the stomach and is then absorbed mainly in the intestine. On the other hand, there are the sublingual and buccal pathways that focus only on the mouth; therefore, they do not include other sites of the gastrointestinal tract. Thus, through the oral cavity is possible to have access to oral, sublingual, buccal, and intragastric routes. Being the oral route is the most used for outpatients as it is safe, easy to use, inexpensive, and painless for the patients. This can help to improve patient compliance, which is very important, particularly in situations where a large part of the population needs to acquire immunity against certain pathogens through vaccination. On the other hand, sometimes, it is necessary to avoid the oral route due to the high enzymatic activity and pH levels. Because acid environments could be detrimental to the stability of certain molecules [1,2], destroying them immediately when in contact with gastric acids. Additionally, sublingual and buccal administration can evade the enterohepatic circulation and thus the resulting first-pass effect of hepatic metabolism [3]. Therefore, using the sublingual and buccal routes, it is possible to avoid these obstacles. Table 1 shows a summary of the characteristics of different administration routes. These routes have become of increasing interest for the administration of a broad group of molecules, for example, those of protein nature. There are numerous peptides and proteins which are used for therapeutic purposes, either to treat a disease or to prevent it. Within the oral cavity, there are two major mucosal delivery sites, the sublingual and buccal areas. These regions, due to their non-keratinized and stratified epithelium, can provide a more elastic and permeable tissue than other regions of the mouth, thus aiding drug absorption. While the hard palate and gingival zones are lined with keratinized stratified epithelium making them more difficult to permeate. These characteristics make them a viable route for drug delivery for small molecules and for vaccines due to the highly developed mucosal-associated immune system [4]

## 2. Mucosal Immune System

Mucous membranes constitute a great and important barrier that protects the body against threats from outside. It represents the first line of defense against viruses, bacteria, and fungi, thus generating an important interaction between the body and the external environment [15].

In fact, about half of the lymphocytes are found in the mucosa-associated lymphoid tissues [16]. Studies in mice have shown that mucosal vaccination against *Helicobacter pylori* can significantly reduce the bacterial colonization in the stomach of infected mice after vaccination [17], demonstrating the efficacy and feasibility of using the mucosal route as an antigen administration route. Furthermore, the administration of antigens through the mucosa can induce local and also systemic immunity. Indeed, some mucosal immune responses appear to be better when the vaccine is administered onto the mucosal surfaces rather than those that have been administered by parenteral injections [11,18,19]. Since the parenteral route is not able to induce effective mucosal immunity, therefore vaccination through mucosal routes would be a better option to achieve effective protection against pathogens. To achieve a successful vaccine delivery to the mucosa, a mucus-permeation strategy is required. The functionalization of nanocarriers offers great advantages when it comes to improving the performance of the formulation and targeting mucin. Among the pathogen’s mechanisms to ease their entrance to the organism, the most common are mucin assembly alteration, degradation, and disruption of the mucus barrier [20]. Functionalization using lectins, glycoconjugates, and microbial ligands such as lipopolysaccharide and flagellin has been one of the ways to overcome the challenge of traversing the mucus barrier. Therefore, mimicking microorganisms approaches have shown favorable outcomes such as the induction of enhanced immune responses in several studies [20,21,22].

This shows that it is very relevant to be able to generate an effective immune response at the level of the different mucous membranes of the organism in order to face the infections that are transmitted through this route and attack the pathogen in time, to prevent infection and with it the disease that is triggered.

MALT is the mucosal-associated lymphoid tissue, and it is able to initiate an immune response against specific antigens present on the mucosal surface [23]. The main function of the MALT is to produce and secrete specific IgA antibodies for antigens along the surface of the mucosa [24]. MALT comprises the immune system related to mucous, which can operate independently of the systemic immune system [24]. MALT is subclassified depending on its location, with nasopharynx-associated lymphoid tissue (NALT), gut-associated lymphoid tissue (GALT), bronchus-associated lymphoid tissue (BALT), and lymphoid tissue associated with the urogenital system being the most important [23,24]. Among the inductive sites found in the mucosal immune system, microfold or M cells play an important role. M cells are mainly found in the GALT and NALT epithelium. Within this role towards immunization, studies have been conducted to target M-cells using LPS [25], FimH [26] and peptidoglycan recognition protein-1 [27]. This use of M-cell-targeting ligands has led to enhanced systemic and mucosal immune responses [28]. Regarding the components of MALT, NALT is one of the major constituents and can induce antigen-specific immune responses [29]. It has been shown that oral-associated lymphoid tissues, especially the nasal-associated lymphoid tissue (NALT), seem to be important for the T-cell activation upon exposure to an antigen. In fact, NALT is one of the first sites that exhibit T-cell activation irrespective of the administration route [30]. Therefore, NALT is one of the main targets of mucosal vaccination to combat mucosal infectious diseases and induce respiratory immune protection [31,32]. Even though the different lymphoid tissues are separated according to their location, are able to remain connected and communicated through the common mucosal immune system (CMIS). This implies that lymphocytes induced by a specific antigen in a mucosal site can migrate to another mucosal site as effector cells to protect tissues from the same antigen in other organs [24,32,33,34,35]. Although there is communication between the mucosal tissues, they are not equal in terms of induction of immune responses. Thereby intranasal vaccination targets the respiratory, gastric and genital tracts, while the oral route is effective for inducing immunity in the gut and mammary tissue, as rectal immunization is useful to elicit colon and rectal immunity and may even confer immunity to the genital tract [36]. This offers the possibility of reaching sites that are complicated to reach through conventional routes. Then it is possible to administer antigens in a mucosal site that is easily accessible, to eventually induce immune responses in mucous membranes distant from the administration site [37].

### 2.1. Comparison between the Parenteral Route; Local vs. Systemic Immunity

Currently, the widest routes used for the administration of antigens are parenteral routes, which are approachable and effective when inducing systemic immune responses. The effectiveness of mucosal routes to achieve an immune response capable of protecting the organism has been demonstrated in a wide variety of studies. These routes have been compared with others, usually parenteral routes since it is the most used nowadays. It is known that it is possible to significantly reduce the transmission of mucosal pathogens through the administration of vaccines at the potential sites of entry of microorganisms. This is due to the ability to generate humoral and cellular responses at the vaccination site [18,37]. Through mucosal immunization, it is possible to induce systemic antibody and cellular immune responses as well as mucosal cytotoxic T CD8+ cell responses (Figure 1) [11,38,39]. In a similar fashion to the parenteral routes, the administration via mucosal has proven to have comparable efficacy. For immunization against simian immunodeficiency virus (SIV) through aerosol showed strong induction of cellular responses in the lungs and systemic humoral immunity becoming as effective as intramuscular [40]. In addition, targeting the respiratory tract mucosal sites could develop more robust mucosal T-cell responses than intramuscular delivery [41,42,43,44]. One key factor in generating this response is the induction of specific immune responses in the mucosal site that is exposed to the threat to interrupt the infection. There are cases where mucosal vaccines have shown greater effectiveness when compared with the same vaccines administered parenterally. In a study carried out in children, with Flumist a live attenuated influenza vaccine, the effectiveness of the same vaccine but administered through two routes, the intranasal and the intramuscular. The results showed that the intranasal route turned out to demonstrate a better protective immunity than in the cases where it was administered intramuscularly [7]. Furthermore, the effectiveness of immunization is closely related to the type of infection to which it is addressed, for example, for protection against pathogens that act in mucosal areas such as influenza virus, rotavirus, and human papillomavirus. The presence of antibodies is required at the entry sites of these pathogens, which are either induced through mucosal immunization, which mainly generates IgA antibodies, or through the transudation of IgG antibodies from the serum generated through the pathways of conventional immunization [45,46]. For infections where immune protection is mainly mediated by the components of the local mucosa, there is impermeability against the transudation of serum antibodies or passive passage through the epithelium. In this case, it would seem the most appropriate to use a pathway of topical mucosal administration [18].

The buccal route for the administration of antigens has generated considerable interest. Since the oral mucosa has a large number of dendritic cells and Langerhans cells, which is remarkably relevant to achieve effective immune responses (Figure 1). Several studies have been published demonstrating the effectiveness of the buccal route. The buccal route of administration is capable of inducing systemic antitumor immunity using a melanoma vaccine in the hamster model [47]. There are studies where orally disintegrating films containing vaccine microparticles have been used, significantly increasing the presentation of antigens and the antibody titers in the juvenile porcine model. Thus, generating an effective immune response through the buccal mucosa [9]. 

Additionally, the oral mucosa was demonstrated to set a systemic antitumor immunity even more efficiently than skin immunization [47], and it showed successful efficacy for distant skin lesions. Another study carried out in mice showed that when administering pDNA through intraoral jet injection in the cheek, the mucosal immune response was achieved by inducing the production of IgA antibodies [8]. The mechanism of infection of the pathogen from which it is intended to protect needs to be considered when choosing the immunization route since it is possible to achieve different responses through different routes using the same antigen. One important topic is the persistence of protection, where T cell memory is the main responsible in the case of a second exposure to the antigen since it generates the quickest response [48,49,50,51]. In addition to the above, the first antigen exposure area is relevant since the permanence of T cell memory will be influenced by this. It has been seen that the recirculation of activated memory lymphocytes is selective and will depend on the tissue and the lymph nodes from where the lymphocytes originated in the first exposure. However, in the case of short-term memory mucosal cytotoxic T lymphocyte memory, the route of immunization is not influenced by the route of administration [52]. This is different from recently activated lymphocytes derived from mucosal tissues or skin because they have shown a preference to migrate back to the sites where the first antigen exposure occurred [52,53,54,55]. Nevertheless, intranasal immunization could result in long-term cytotoxic T lymphocyte memory in the local mucosa of the respiratory tract, but also distant tissues such as the genital tract [11]. The above would realize that the genital tract is an implicit effector site within the common mucosal immune system. It has been shown that in the case of long-term responses, when the administration is through the nasal mucosa, the mucosal memory is long-lasting while the systemic memory is short. In contrast to parenteral immunization, where mucosal memory is short and systemic is durable [11]. Using the sublingual route to administer vaccines, it is even possible to achieve long-term immune responses through the efficient induction of antigen-specific long-lived plasma cells [10]. Where the immune response generated is only due to the participation of the sublingual mucosa, and when administering the same dose orally, this response is not observed [10].

### 2.2. Induction of Effective Immune Responses at Sites Distant from the Site of Administration

The fact that the mucous membranes maintain communication with each other despite being physically distant is interesting when considering administering antigens that require their action at distant sites. In a study carried out in mice, an HIV subunit vaccine was administered through the intranasal, sublingual, and intravaginal routes. In which the intravaginal route was effective in inducing antibodies in the genital mucosa area [12]. In fact is possible to achieve distal humoral responses in the vaginal and rectal mucosa due to the targeting towards the mucosal sites located in the respiratory tract [40]. However, the effectiveness of immunization is highly influenced by the hormonal fluctuations of the individual [12]. Regarding this, it has been seen that estradiol is responsible for the variable effectiveness of the intravaginal route since it has been observed that it inhibits CD8+ T cell priming [56]. Furthermore, both the intravaginal and intrarectal routes, despite being effective in inducing an immune response, have poor patient compliance [18].

As alternatives to the intravaginal route, the sublingual and intranasal routes were tested, which were found to have similar effectiveness. However, the intranasal route has generated certain alerts regarding its safety since unwanted adverse effects related to damage to the central nervous system have been reported. Among these complications, adenovirus translocation to the olfactory bulb in mice, Bell’s Palsy have been described in volunteers who have been immunized against the influenza virus through the intranasal route [57]. Additionally, it has also been reported that the LTK63 delivery through the intranasal route in healthy humans produced toxic effects [13]. It is believed that there is a retrograde passage of components of the formulation towards the central nervous system after ganglioside binding, which is not observed through the sublingual route. It is believed that there is a retrograde passage of components from the formulation towards the central nervous system after ganglioside binding [13], which is not observed through the sublingual route [57,58,59,60,61,62,63].

### 2.3. Sublingual Delivery of Antigens as an Alternative for Allergy Treatment

The mucosal surface, as it is the first barrier against pathogens, must-have defense mechanisms capable of protecting the organism from the external environment. However, simultaneously also need to acquire tolerance against non-dangerous inhaled or orally absorbed antigens and maintain the local microbiota in balance [64]. However, when these mechanisms do not work properly, allergies to common elements in the environment can occur, as with some components of food. The mucosal routes have not only proven to be useful and effective in protecting against pathogens, but they are also interesting for the treatment of certain allergies. Allergies are a problem that affects a significant part of the population, from young children to older adults. The most common allergen that causes airway problems is grass pollen [65,66]. This allergy frequently manifests itself with rhinitis or even asthma and is generally treated with antihistamines and corticosteroids to control the symptoms, but that does not improve the problem from the causes. One of the treatments that could help solve the cause of the problem is specific immunotherapy [67], which can be administered through the subcutaneous route. As an alternative to conventional routes, sublingual has been proposed since the administration of antigens has proven to be effective for suppressing IgG and IgE antibody responses, essential in allergy treatments [68,69].

The use of the sublingual route proposes a non-invasive, painless, and safe treatment since fatal cases associated with sublingual immunotherapy have never been reported [58]. In fact, the use of alternative routes such as the sublingual route could become safer by minimizing the risk of inducing anaphylaxis during therapy [6]. This turns out to be an important advantage in terms of the safety of the sublingual route over other conventionally used routes. In addition, the sublingual route in the administration of OVA as an allergen was shown to induce a higher IgE antibody suppression than when it was administered intragastrically [70]. As has been shown, the sublingual route as an allergen administration route could become safer and even more effective in some cases for the specific treatment of allergies.

As has been observed in the studies shown above, the administration of antigens through the mucosal routes can be beneficial in several circumstances. They could be useful, for example, in pediatric or geriatric patients with swallowing problems. In addition, in some cases, the fact of administering the antigens through the mucosal route is observed in greater effectiveness in the treatment compared to other routes commonly used.

## 3. Antigen Delivery Systems and Commonly Used Mucosal Adjuvants

The oral cavity shows great potential for antigen delivery. Various dosage forms have been developed that use the oral cavity as the site of administration, with tablets [71], mucoadhesive films [5,6,71,72,73], gels [74], microneedles [75,76,77,78], and MucoJet™ [79] being reported (Table 2). The routes that comprise the oral cavity can be beneficial in many cases, increasing patient compliance because of its ease of administration and allowing a large-scale immunization. However, it could exhibit some difficulties (Table 1). Among the challenges that delivery systems face is the need to maintain sufficient concentrations within the administration area, since physiologically, there is a wash due to the anatomy of the oral cavity. The movement of the cheeks, the tongue, and the amount of saliva could make the administration more difficult and alter the concentrations of the drugs or biologics administered. This could even detach the film or tablet that should be adhered to the tissue.

### 3.1. Films

As mentioned above, the oral cavity is a site of great interest for the administration of antigens. Immunization in its vast majority consists of an administration through an injection, mainly intramuscular or subcutaneous, which on many occasions can hinder the process. Through the oral cavity, it is possible to deliver a wide variety of dosage forms, including films. Although the buccal and sublingual routes exhibit some disadvantages, such as motion stress or salivary washout, certain properties of films are able to overcome these obstacles. The main characteristic of mucoadhesive films is to be able to remain on the surface of the oral cavity long enough to release the content into the buccal mucosa. There are several methods to manufacture films; within these, the simplest is by solvent casting method. In this procedure, the active pharmaceutical ingredient can be incorporated into the gel solution, and then when it dries, a film is formed which contains the antigen inside or otherwise. It is possible to load the film at the end of the manufacturing process. The development of buccal films has allowed the manufacture of films in bilayers or multilayers; in this way, there can be layers with different functions, thus optimizing the delivery system.

**Figure 2:**
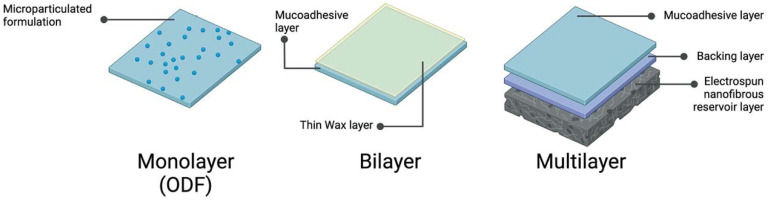
Different types of oral mucosa delivery film systems and each of its layers.

Referring to the manufacture of monolayer films, orally dissolving films (ODF) have proven to be of great interest. Not only in the application of a wide variety of drugs but also in the delivery of vaccines through the oral cavity. This type of film dissolves quickly when it comes into contact with saliva, so it is comfortable for the patient and reduces the risk of choking. A group of researchers manufactured a novel oral measles vaccine [9]. Which consists of an ODF, where the vaccine to be administered is incorporated into the film in the form of microparticles manufactured by spray drying. The components of the gel that will form the film, Lycoat RS720^®^, Neosorb P60W^®^, and Tween 80 will result in an enteric polymeric matrix (Figure 2). One of the advantages that this matrix has is to optimize the absorption of swallowed particles that were not absorbed in the oral cavity. Since they could reach the intestine in good condition where they could generate an additional immune response, in this study, in vivo immunity tests were carried out using the juvenile porcine model. Since the human mucosa is non-keratinized and there is also a great resemblance between the structure and composition [80] of the porcine and human mucosa, making this model is quite accurate. An initial dose of ODF was administered, and then after two weeks, a booster dose was observed that this formulation is capable of inducing a significant immune response. Herein the presentation of antigens to the MHC I and MHC II molecules was increased, compared to the formulation that did not contain the vaccine. Furthermore, even higher antibody titers were observed at weeks four and six, wherein compared with the levels prior to immunization [9].

Regarding the films composed of a bilayer, Cui and Mumper [5] carried out an investigation where the formulation of mucoadhesive films was composed of two layers. A thin layer of wax and another mucoadhesive layer contains the antigen either inside or on the surface (Figure 2). The mucoadhesive layer was manufactured using a mixture of two polymers, Noveon AA-1, a cross-linked mucoadhesive polyacrylate polymer, and Eudragit S-100, an anionic pH-sensitive co-polymer of polymethacrylic acid-co-methyl methacrylate. Briefly, the method of manufacturing the films consists of preparing an ethanol-based gel and then drying it to form the film. Finally, one side of the film is embedded in melt wax to give it a second layer which is thought to help retard the diffusion. Although this variable has not been studied in this research. β-galactosidase or plasmid DNA-expressing β-galactosidase was used as a protein antigen model. Regarding the loading of the antigen, two methods were used. The preloading where the antigen was placed on the gel before drying it and the post-loading, where the antigen was loaded once the film had already formed. The latter showed the best release, 60% in 2 h for pDNa and 80% for β-galactosidase. From this study, it was found that there is an optimal ratio to achieve greater mucoadherence, where the 3:1 Noveon/Eudragit composition demonstrated the strongest mucoadherence. Besides investigating the proportion of polymers to achieve good mucoadhesion, in vivo tests were carried out in rabbits to evaluate immune responses. When analyzing the results of mucosal immunization compared to subcutaneous immunization in rabbits, it showed that it was possible to induce antigen-specific IgG through mucoadhesive films. At 28 days after immunization, IgG titers were significantly greater in rabbits immunized by the buccal route compared to the subcutaneous route. Additionally, it was possible to demonstrate that rabbits that were immunized with buccal bilayer films containing pDNA developed signs of cellular immunity since it was possible to observe positive splenocyte proliferative responses [5].

The development of buccal and sublingual films has made it possible to manufacture films with three or even more layers, to which different characteristics can be attributed in order to achieve successful administration. Due to the technology behind these multilayer systems, the release and delivery of the antigen can be controlled in order to maintain a long-term concentration gradient, preventing it from being rapidly eliminated by saliva. In addition, the incorporation of a waterproof backing layer, as in the previous case of bilayer films, makes it possible for the movement of the film content to be unidirectional. Since the side that gets contact with the rest of the oral cavity is unable to release the content of the film. About obtaining a large surface-to-weight ratio, the technologies of the advantages of multilayers, nanoparticles, and nanofibers have been combined [72]. Mašek et al. Manufactured multilayer films for the administration of vaccination nanoparticles; these delivery systems consisted of three layers, a mucoadhesive, a backing, and an electrospun nanofibrous reservoir layer (Figure 2). The mucoadhesive layer was prepared, which was covered with Eudragit L 100-55 to form the backing layer. Finally, the surface of the mucoadhesive layer was moistened and pressed against the nanofibrous reservoir layer to achieve its adhesion. Each of these layers fulfills a necessary function to achieve an appropriate administration. The mucoadhesive layer, as seen above, is necessary to fix the film on the oral mucosa and allow it to remain adhered for as long as necessary. The function of the oral dissolving backing layer is to protect the integrity of the mucoadhesive film and for the comfort of the patient since it does not have to be removed. Finally, the nanofibrous layer has a more complex technology and structure than the other two layers, and the release of its content will depend on the materials used in the formulation, making it faster or more controlled. Due to its extremely high surface area and the channels formed between the fibers, which give it high porosity, it can store a large amount of content. These differences mean that it has advantages over layers composed of other materials such as dried gels; since they are simpler, its storage capacity is reduced. Nanofiber layers were made with three different biocompatible materials; Silk fibroin (SF), Chitosan-Polyethylene oxide (Chitosan-PEO), Polycaprolactone (PCL). Wherein one of the most important factors to study for this layer is the degree of adhesiveness of the material that has to do with the hydrophilicity of the layers. Generally, materials like Chitosan-PEO, SF, and PCL pretreated with NaOH, can provide high hydrophilicity, which is related to the releasability of nanoparticles. It has been observed that films with hydrophilic surfaces were able to release about all the necessary content during the given incubation time. In addition, liposomes and nanoparticles with fluorescent markers were also manufactured and were later incorporated into the nanofibrous layer. Ex vivo studies were conducted in oral mucosa porcine where multilayer formulations were compared with free nanoparticles. It has been shown that films are able to maintain their fluorescence for an extended time at the site of administration, while free nanoparticles were rapidly washed out of the mucosal surface and were not able to penetrate through the mucosa. In addition, in vivo studies were carried out in piglets to which a multi-layered mucoadhesive film was applied to the sublingual mucosa. The nanofiber layer was SF, and the content of these films were fluorescently labeled Poly (lactic acid-co-glycolic acid and polyethylene glycol (PLGA-PEG) nanoparticles, wherein the PEG fulfills the role of providing the particles muco-penetrating features [81]. When analyzing the results, the presence of fluorescent nanoparticles in porcine antigen-presenting cells (pAPC) was found in the lymph nodes. Therefore, this delivery system is interesting for oral or sublingual immunization due to its potential to maintain high concentrations at the administration site. Additionally, to go across the oral mucosa, reaching sites of interest such as lymph nodes.

Mucoadhesive films are a versatile platform for the administration of drugs in different ways, including biological and particulate systems. It is also possible to modify the characteristics of the films and thus achieve control of the release from the delivery system. Its manufacturing complexity will depend on the method used; the elaboration difficulty can range from the dehydration of a gel to the manufacture of an electrospun nanofiber system. Films have the great advantage that they can be modifiable in terms of structure, composition, size, hydrophilicity, thickness, mucoadhesiveness, solubility, among others. All these characteristics make the films highly interesting drug delivery systems in the field of mucosal vaccination.

### 3.2. Microneedle Array

**Figure 3:**
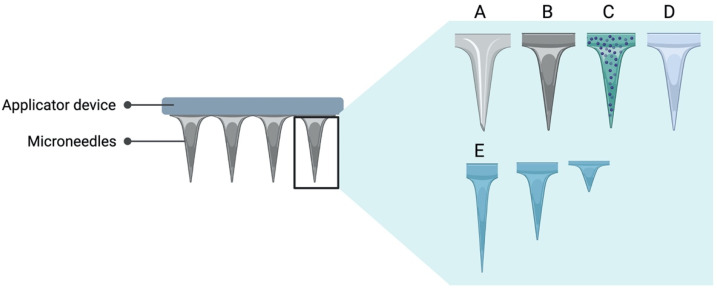
Representation of a variety of microneedles. Shape, size, and materials are modifiable. The tip of the microneedles can be (**A**) hollow, (**B**) solid, (**C**) coated, or (**D**) dissolvable. (**E**) The depth can be modifiable through the geometry and sharpness of the tip.

Microneedles have been extensively studied for intradermic vaccine delivery; however, studies involving the oral cavity are limited. Similar to films, microneedle delivery systems are painless and comfortable for the patient, easy to place without the need for trained personnel, unlike routes that require puncture. Microneedles also have the advantage that they are highly changeable and can have control of various parameters when delivery as depth, uniformity, and dose to be administered. The geometry of the microneedle tip is one of the key parameters when it comes to studying its skin-penetrating ability (Figure 3). The sharpness and the pitch angle of the microneedle tip can impact the amount of content deposited into the skin [82]. It is also possible to modify the characteristics of the tips; they can be solid, hollow, coated, or dissolvable (Figure 3). In terms of content, there are formulations that include particulate systems either as coverage of the tips or inside if hollow. The content of the coverage of the tips is highly modifiable. 

There are several sorts of compounds that have been used as coverage of microneedles, such as viral particles, from bacteria, DNA plasmids, and so on [83,84]. Regarding the development of coated microneedles, it has been shown that these delivery systems could induce immune responses when administered into the oral cavity. Ma et al. [75] manufactured microneedles coated with three types of antigens, two HIV antigens, and ovalbumin. These microneedles were administered on the inner lip and the dorsal surface of the tongue of rabbits to evaluate the immune response. The structure of the microneedles was fabricated from 50 µm-thick stainless-steel sheet to which different coatings were subsequently placed. The base of the coating solution was made of carboxymethylcellulose sodium salt and Lutrol F-68 NF, then sulforhodamine or ovalbumin or E2V3 or DNA expressing gp160 was added. The last three were the antigens studied, while the first was used as a dye to assess penetration into the surface of the oral cavity of rabbits. After inserting the microneedles for 2 min, the delivery efficiency of microneedle coatings was observed, wherein the lip, it was about 63.0% and for the tongue, close to 91.2%. These values turned out to be quite similar to what has been studied previously for administrations on the surface of the skin, which has been reported from 50% to 90% [83,85,86]. Regarding delivery efficiency, it can be mentioned that it is a parameter that is closely related to the nature of the materials, specifically to their hydrophilicity. It has been seen in subsequent studies that for hydrophilic coatings, the delivery efficiency is much higher in both human and mouse skin models compared to coatings of a hydrophobic nature [76,86]. Another parameter to consider is the thickness of the coating, which has been related to a lower delivery efficiency [86]. The similarity obtained between the percentage of delivery for the oral cavity and for the skin turns out to be an interesting result. Despite the unfavorable conditions that may exist inside the oral cavity, such as the lack of bone structure and the flow of saliva, delivery via microneedles confers the right delivery efficiency. Despite the differences between the inner lip and the dorsal surface of the tongue, the immune responses against ovalbumin were quite similar. Both sites were equally immunogenic and suitable for the administration of antigens, generating an immune response after the second dose was administered. In this study, the ability of the mucosal route to induce better responses than the intramuscular route is once again demonstrated for immunization against HIV. The systemic response generated by the IM route was similar to that of the mucosal route, resulting in similar levels of IgG. However, when comparing the immune response in saliva, it is observed that the IgA levels reached by the mucosal route were higher than the IM route, where the immune response was quite weak. In another study where ovalbumin was also used as antigen, coated microneedles were prepared with OVA and with OVA plus Cholera Toxin (CT) as an adjuvant, and disks without tips. Then IgG levels were evaluated after two weeks of the second administration of these delivery systems [76]. In this case, the matrix had 13 needles and both the microneedle arrays, and the disks were prepared by micromillling and the replicas by micromolding. Both structures are made based on polylactic acid (PLA), and the coatings were manufactured based on carboxymethyl cellulose (CMC), to which OVA and CT were subsequently added if applicable. In this case, the PLA base acts as a backing layer to support the system. It has been shown that all the microneedles were successfully inserted into the mucosa and that the coating was able to dissolve inside it due to the high solubility of the main component. The distribution of OVA plus a fluorescent marker in porcine mucosa was studied. When comparing the results of the microneedles and the disks, it was obtained that at 20 min, the microneedles had already diffused towards the mucosa. On the other hand, in the case of disks, it occurred very slowly, which indicates that it must be in contact with the surface for several hours to deliver its content via transmucosal. When performing in vivo vaccination, the devices were attached for 20 min, and then the immune responses were evaluated. In the case of disks, no effective response is observed at the mucosal level, most likely since it takes more time to release the content. On the other hand, for the microneedle arrays, a greater response was obtained when compared with that induced by the disks. Furthermore, when comparing the response induced by the microneedle array containing CT as an adjuvant, the increase in IgG titers was even greater. 

With dissolving microneedles, it is possible to include lysosomes within the formulation to be a building block of the micron size needle structure which aids the delivery into the mucous membranes and dissolves locally to release the antigen. Recently, this unique type of antigen delivery system has been tested for the oral cavity. Zhen et al. describe a BSA that was used as an antigen model, and the microneedle array was manufactured based on MPC/lipid-A liposomes. These microneedles were manufactured by a procedure of emulsification-lyophilization where an inverse mold was subsequently used to form the array of 6 × 6 structure of the microneedles [77]. The most important attribute for dissolving microneedles, they must be sufficiently hard to penetrate the site of administration, which was achieved in this study by a matrix of sucrose and PVP. Once again, conventional and non-conventional routes of administration were compared; in this case, it is observed that through the subcutaneous route, it was not possible to induce a mucosal immune response, unlike immunization by means of microneedles administered in the oral cavity where high levels of IgA were present in saliva, intestinal and vaginal washes. These parameters were also compared with the free liposomes, where a much lower response is observed than compared to the pro-MPC/lipid-A liposome-filled microneedle array (proMMAs). The microneedle applicator device can help as a base since, unlike the other areas of the skin, the mucosa lacks muscles or bones that can provide support. Application systems are used in order to improve delivery efficiency and reduce variations that may exist in users with little experience in inserting the device [87].

Most vaccines administered by conventional routes require cold chains, which makes distribution and transport challenging. Through the microneedles, it is possible to lower the temperature requirements to maintain stability. In the case of MLLs, the results of the stability studies showed that after 4 °C for six months, 25 °C for two weeks, or at 40 °C for three days they remained stable [77]. Only slight variations are observed in size and zeta potential, while when analyzing the integrity of the model antigen, it has been shown that there was no significant degradation at 4 °C for 180 days, 25 °C for 14 days, and 40 °C for three days [77]. This indicates that these microneedle arrays can be kept under a controlled temperature chain. These do not require high energy consumption, unlike parenteral vaccines that must be stored at very low temperatures. This is an advantage for distribution and transportation logistics, which lowers costs and gives greater accessibility to immunization in regions where it is not possible to maintain extremely cold chains.

Microneedle systems definitely have great potential to be used as means of immunization against pathogens that affect the mucosal tract. Given the broad advantages these delivery systems own, they are stable at room temperature [77,78,88,89,90], easy to use, painless and comfortable [91] for the patient, and do not require trained personnel. All these features make them useful when considering mass immunization in a short period of time as required in times of pandemics. Definitely, immunization using microneedles in the oral cavity is a field in which many details are still unknown. The vast majority of studies related to microneedling vaccine delivery use the skin as the site of administration, and only a few have focused on the oral cavity. Although the information on this specific topic is limited, the results obtained by the investigations have been quite favorable and encouraging. A summary of the mentioned studies regarding films and microneedle array systems is given in Table 2. In the future, it could be interesting to expand the scope of cutaneous administration to the mucosal routes. Besides, in this way, it may even be possible to obtain a more robust immune response.

### 3.3. Mucosal Adjuvants

Tolerance is an obstacle that the oral cavity presents as a site for antigen administration, which is why many formulations end up being ineffective when it comes to inducing immune responses. Co-administration of an enhancer agent of the immune response as are adjuvants helps potentiate the immunogenicity of the formulation. Although there are quite effective compounds, they are usually dangerous for use in humans since many proceed from bacterial toxins and cause notable toxicities. The development of safe and effective mucosal adjuvants could be just as important as the development of antigens since both are necessary for effective and safe immunization. All adjuvants presented below are summarized in Table 3.

#### 3.3.1. Bacterial Enterotoxins

Regarding the adjuvants from pathogenic bacteria, the cholera toxin is the primary enterotoxin produced by the bacterium *Vibrio cholerae*. This toxin is composed of five binding subunits (B) and one toxic-active A subunit. CT acts by increasing the presentation of antigens by macrophages, enterocytes, and B cells, which is relevant within mucosal adjuvants since it has been shown to have great potency [92]. However, despite being effective, it presents significant toxicity in humans, generating enterotoxicity and severe diarrhea [93]. Accumulations in the CNS associated with neurological side effects have also been reported [57]. Yet, it is still widely used in animal models to evaluate the immune responses of formulations. In antigen administration where CT was used as an adjuvant, it was observed that there were no adverse effects either through buccal films or by subcutaneous injections in rabbits [5]. In order to separate adjuvant activity and enterotoxicity, non-toxic CT subunits are used. An example of the above is the adjuvant CTA1-DD which is completely non-toxic and safe. Doses of 200 µg administered in mice and monkeys did not present apparent toxic effects, is that the same dose of CT would be lethal [99]. Regarding the administration through the oral cavity, the effectiveness of immunization has been evaluated in an oral microneedle system, in which the immunogenicity of the OVA formulation with and without adjuvant was evaluated. It is observed that the administration system is able to generate a mild immune response without adjuvant; however, when compared with that generated by the formulation containing CT, this induction of the immune system was much higher. It is also observed that this formulation of microneedles with OVA plus CT is capable of generating both systemic and local immune responses [76]. As well as the cholera toxin, there is also the heat-labile enterotoxin of Escherichia coli (LT), which has immunomodulatory properties similar to those of CT and has similar potential as an adjuvant [94]. But like CT, it causes severe diarrhea, so the native form is not suitable for use as a mucosal adjuvant in humans. Several LT mutations have been developed in order to reduce the toxic effects; however, some of them, in addition to losing the toxicity, also lost the adjuvant effect. However, not all LT mutations are free of adverse effects; the single-mutant LT (LTR192G) was used in a clinical study [100] that involved vaccination against *Helicobacter pylori* through the oral route. It turned out that some volunteers had episodes of diarrhea that, in some cases, lasted for up to three days. This side effect was observed only in subjects to whom mLT was administered, whether or not in association with the vaccine under study. While all the volunteers presented at least one gastrointestinal effect, which can be attributed to both the vaccine and the adjuvant. However, this complication is not the first time that it occurred in a clinical trial; native LT was used as an adjuvant again for a vaccine against *H. pylori*. The incidence of diarrhea in volunteers was quite high, and in some of them, this complication was such that it affected their daily activities. Consequently, the amount of LT administered had to be cut in half [101]. The development of LT variations such as double-mutant heat-labile toxin (dmLT) has demonstrated strong mucosal immune responses in mice [95], where the immune response against *H. pylori* was evaluated after sublingual immunization. It turned out that dmLT and CT presented similar potencies as an adjuvant, acting mainly in the cervical lymph nodes after sublingual administration. However, when comparing the response observed in the mesenteric lymph node at the same dose but in intragastric administration, it was much lower. Regarding dmLT, there are already Phase 1 clinical studies where this adjuvant has been used in oral vaccinations. The safety and tolerability of the vaccine used without the adjuvant, with 10 µg or 20 µg of dmLT, was tested. The results showed that the vaccine in these three forms turned out to be safe and well-tolerated by the volunteers [102]. Only three volunteers out of a total of 129 had diarrhea, one of them was not attributed to the vaccine since they had only received a placebo. In the latter study, it reported that adding dmLT to the vaccine formulation did not alter the safety profile.

#### 3.3.2. Toll-like Receptor Agonist

Another adjuvant is the CpG Oligodeoxynucleotide, which consists of a small synthetic DNA chain that has been shown to have great immunostimulatory activity when administered parenterally but also by mucosal route [103,104]. The action of synthetic CpG is to mimic a stimulation by bacterial infection and then interact with Toll-like Receptor 9 (TLR-9) [96]. This can be seen in TLR9-knockout mice, in which it is not possible to observe any immune response after the administration of CpG. In a study conducted where sublingual vaccination against *Porphyromas gingivalis* was performed using CpG as adjuvant [105]. It was determined that the use of CpG in the formulation resulted in more potent immune responses. When CpG was added to the formulation, a high concentration of IgG and IgA was observed in the spleen and saliva, respectively. Whereas when adjuvant was not administered, the levels of antibodies and antibody-forming cells (AFC) remained undetectable. Regarding the potency of CpG, it has been seen that synergy with CT is generated. When applied simultaneously, their activity increases significantly, achieving greater responses than those compared with ten times each adjuvant separately [104].

#### 3.3.3. Polymers

Chitosan is a cationic polysaccharide that has great biological compatibility and is also biodegradable. Chitosan has many uses; among them, it is capable of improving paracellular transport through the epithelium, which is very useful when considering enhancing the absorption of antigens administered by mucosal routes. There are researchers that agree with this information since an increase in the transport and penetration of molecules was demonstrated in the presence of chitosan through pathways that involve mucosa [106,107,108]. The mechanism of action is thought that through the protection of the antigen, it achieves a greater uptake and presentation, which leads to an increased immune response [109,110,111]. Chitosan has also been used for the manufacture of microneedles, where it would have various uses, as a structure, as a mucosal adjuvant, and achieve a prolonged release. Another use that has been given to chitosan is the manufacture of micro and nanocarriers for the delivery of vaccines [97]. Chitosan has also been used for the manufacture of multilayered mucoadhesive films where the formulations composed of chitosan were able to release almost all the adsorbed particles in vitro [72].

Among the most important barriers that exist in the oral cavity and in many others is the mucosal barrier, which fulfills the function of protecting the organism from exogenous threats. For this reason, it is often difficult to achieve effective immunization in these areas. In order to overcome this obstacle, particles with muco-penetrating characteristics have been used, which help to diffuse rapidly through the mucus. To achieve producing particles with these characteristics, a Polyethylene glycol (PEG) coating can confer the ability to cross the mucus barrier. However, in order to achieve it successfully, the lining must be sufficiently dense; otherwise, the particles may remain trapped in the mucus, slowing down its penetration [81]. PEG has a dual action, where it can provide mucoadhesive or mucopenetrant properties depending mainly on the density of the coating and the molecular weight. When the coating is dense with low molecular weight PEG, it leads to rapid mucus penetration. While the opposite occurs when high molecular weight PEG is used, mucoadhesion is increased since intermolecular interactions such as hydrogen bonds with mucins. PEG has been widely used in pharmaceutical development focused on the administration of proteins and nanoparticles since it increases their half-life by reducing interactions with serum proteins and with macrophages [98,112]. In relation to the administration of antigens, PEG has been used to coat nanoparticles that are inside a film to be used as oral and sublingual immunization [72]. In this case, a film was designed in which one of the layers had mucoadhesive characteristics using HPMC and Carbopol, while the reservoir layer contained mucopenetrating nanoparticles. Both strategies work together to achieve enhanced oromucosal delivery. In addition to presenting mucopenetrating characteristics, the inclusion of PEG in the formulation could help enhance lymph node delivery of nanoparticles, which is quite interesting when formulating a mucosal vaccine [98]. The degree of PEGylation can influence the targeting of several cells, such as migrating DCs, and can lead to a more robust immune response if the access of vaccines to DCs is carried out. PEGylation is known to influence draining lymph node transport and accumulation of antigens [113]; nevertheless, a completely correct mechanism has not been demonstrated. Although it has been demonstrated that when administering PEGylated particles, the presentation of antigens and T cell priming has been increased, in addition to presenting a targeting towards lymph nodes [114]. However, the mechanism PEG is not completely clear since there are many factors that influence the lymphatic trafficking of particles, and the results of the studies have not been entirely accurate. PEG undoubtedly has many uses in the pharmaceutical industry and has great potential in the administration of antigens through the mucosal route. Because of all the properties that it can confer to the particles in the field of immunization.

## 4. Conclusions

Immunization through unconventional routes such as oral and sublingual routes has been shown great potential accompanied by an outstanding efficacy by other parenteral routes. It is extremely important to develop vaccine formulations that are administered through mucosal routes since they would be very useful nowadays. Undoubtedly, we face a different world, where pathogens that access the body through mucosal routes present a great risk for the world population. Strengthening the mucosal immune system suggests additional protection against these threats. Beyond how effective mucosal vaccines could be, many other advantages related to the transport and manufacture of doses also stand out. Currently, achieving a mass and effective immunization program against SARS-CoV2 has been one of the greatest challenges of modern times. The storage and transport of the doses require extensive cold storage supply chains that are difficult to maintain, so the costs associated with vaccination are increased. Thus, hindering the access to affordable vaccines for many low-income countries with limited cold chain logistics, leading to suboptimal immunizations. Vaccine development in oral dosage forms such as films [73,115] and microneedle arrays [77] have been shown to maintain its stability even under extreme conditions. These systems could be loaded with antigen particles that are found as dry powder. Therefore, being in a solid-state, it would not require freezer or refrigerator systems [116], which would facilitate both the storage and the transport of the doses. The needle-free self-immunization accroach allows for mass immunizations and improves patient compliance multi-fold. All these factors are beneficial in the context of mass vaccination; these delivery systems propose multiple benefits for the patient. Such as ease of administration at all ages, comfort, painless application, and there is no risk of choking; therefore, it can even be self-administrated. Moreover, the fact that they are needle-free leads to a lower risk of contamination by biological waste. Self-immunization can also be beneficial in times of pandemic when mass immunization is required; it can help reduce the burden on the healthcare system, especially for front-line workers. These vaccine delivery systems could help to improve patient compliance compared to conventional administrations. Undoubtedly, due to the fact that they have shown great potential, these technologies could revolutionize how the population gets vaccinated, leading to a more economical and comfortable immunization process.

## Figures and Tables

**Table 1:** Comparison between routes of administration.

Route of Administration	Advantage	Disadvantage	Reference
Subcutaneous	Assured absorption	Pain at the puncture site	[5]
	Induce systemic immune responses	Requires medical personnel	[6]
	Avoid first-pass effect	Does not induce effective mucosal immune responses	
Intramuscular	Assured adsorption	Pain at the puncture site	[7]
	Induce systemic immune responses Avoid first-pass effect	Requires medical personnel Does not induce effective mucosal immune responses Requires cold chain Expensive to prepare	[6]
Oral	Painless	Degradation of vaccine by harsh stomach environment and high enzymatic levels	[8]
	Induce systemic and mucosal immune responses	Taste issues	[6]
	Easy to administer		
Buccal	Painless Induce systemic and mucosal immune responses	Salivary washout can dilute the vaccine Motion stress Taste issues	[5,9]
	Easy to administer Mild pH values		[6]
Sublingual	Painless Induce systemic and mucosal immune responses Easy	Salivary washout can dilute the vaccine Motion stress Taste issues	[6,10]
Intranasal	Painless Induce systemic and mucosal immune responses Mild pH values	Retrograde passage to the CNS Short residence time Quick clearance of antigens	[7,11,12,13,14]

**Table 2:** Characteristics of antigen delivery systems.

Administration Route	Dosage Form	Main Feature	Antigen/Model Antigen Used	Reference
Buccal	Film	Orally disintegrating film loaded with microparticulate vaccine	Live attenuated Measles microparticulate vaccine	[9]
Buccal	Film	Bilayer mucoadhesive film	β-galactosidase/plasmid DNA-expressing β-galactosidase	[5]
Buccal	Film	Electrospun nanofibrous reservoir multilayer film	Green fluorescent protein loaded nanoparticle and liposomes	[72]
Inner lip/ Tongue	Microneedle	Solid stainless steel coated microneedle array	HIV and Ovalbumin Antigens	[75]
Buccal	Microneedle	CMC coated solid PLA micromeedle array	Ovalbumin	[76]
Oral mucosa	Microneedle	Liposome loaded dissolving microneedle array	BSA	[77]

**Table 3:** A compilation of reported molecules used as mucosal adjuvants.

Adjuvant	Origin	Effect	Reference
Cholera Toxin	*Vibrio cholera*	Immunostimulatory	[92]
CTA1-DD	*Vibrio cholera*	Immunostimulatory	[93]
Heat-labile enterotoxin LT	*Escherichia coli*	Immunostimulatory	[94]
DmLT	*Escherichia coli*	Immunostimulatory	[95]
CpG DNA	Oligonucleotide DNA	Immunostimulatory	[96]
Chitosan	Cationic polysaccharide	Enhance adsorption of antigens	[97]
Polyethylene Glycol PEG	Polymeric	Mucoadhesion and mucopenetration	[98]

## Abbreviations

MALT	mucosa-associated lymphoid tissue
NALT	nasopharynx-associated lymphoid tissue
BALT	bronchus-associated lymphoid tissue
GALT	gut-associated lymphoid tissue
CMIS	common mucosal immune system
SIV	simian immunodeficiency virus
Ig	immunoglobulin
HIV	human immunodeficiency virus
OVA	ovalbumin
ODF	orally dissolving films
MHC	major histocompatibility complex
SF	silk fibroin
PCL	polycaprolactone
Chitosan-PEO	chitosan-polyethylene oxide
PLGA-PEG	poly(lactic acid-co-glycolic acid and polyethylene glycol
pAPC	porcine antigen-presenting cells
CT	cholera toxin
PLA	polylactic acid
CMC	carboxymethyl cellulose
BSA	bovine serum albumin
MPC	mannose-PEG-cholesterol
PVP	polyvinylpyrrolidone
MLL	MPC/lipid A-liposome
proMMA	proMLL-filled microneedle array
CNS	central nervous system
LT	lethal toxin
mLT	mutant lethal toxin
dmLT	double mutant lethal toxin
AFC	antibody-forming cells
PEG	polyethylene glycol
HPMC	hydroxypropyl methylcellulose
DCs	Dendritic cells.
